# Bottom-up proteomics analysis for adduction of the broad-spectrum herbicide atrazine to histone

**DOI:** 10.1007/s00216-023-04545-6

**Published:** 2023-01-20

**Authors:** Shaogang Chu, Robert J. Letcher

**Affiliations:** Ecotoxicology and Wildlife Health Division, Wildlife and Landscape Science Directorate, Environment and Climate Change Canada, National Wildlife Research Centre, Carleton University, 1125 Colonel By Drive, Ottawa, ON K1A 0H3 Canada

**Keywords:** Histone, Atrazine, Adduction, Post-translational modification, UHPLC, High-resolution Orbitrap mass spectrometry

## Abstract

Histones are the major proteinaceous components of chromatin in eukaryotic cells and an important part of the epigenome. The broad-spectrum herbicide atrazine (2-chloro-4-[ethylamino]-6-[isopropylamino]-1, 3, 5-triazine) and its metabolites are known to form protein adducts, but the formation of atrazine–histone adducts has not been studied. In this study, a bottom-up proteomics analysis method was optimized and applied to identify histone adduction by atrazine in vitro*.* Whole histones of calf thymus or human histone H3.3 were incubated with atrazine. After solvent-based protein precipitation, the protein was digested by trypsin/Glu-C and the resulting peptides were analyzed by high-resolution mass spectrometry using an ultra-high-performance liquid chromatograph interfaced with a quadrupole Exactive-Orbitrap mass spectrometer. The resulting tryptic/Glu-C peptide of DTNLCAIHAK from calf thymus histone H3.1 or human histone H3.3 was identified with an accurate mass shift of  +179.117 Da in atrazine incubated samples. It is deduced that a chemical group with an elemental composition of C_8_H_13_N_5_ (179.1171 Da) from atrazine adducted with calf thymus histone H3.1 or human histone H3.3. It was confirmed by MS/MS analysis that the adduction position was at its cysteine 110 residue. Time- and concentration-dependent assays also confirmed the non-enzymatic covalent modification of histone H3.3 by atrazine in vitro. Thus, the potential exists that atrazine adduction may lead to the alteration of histones that subsequently disturbs their normal function.

**Graphical abstract:**

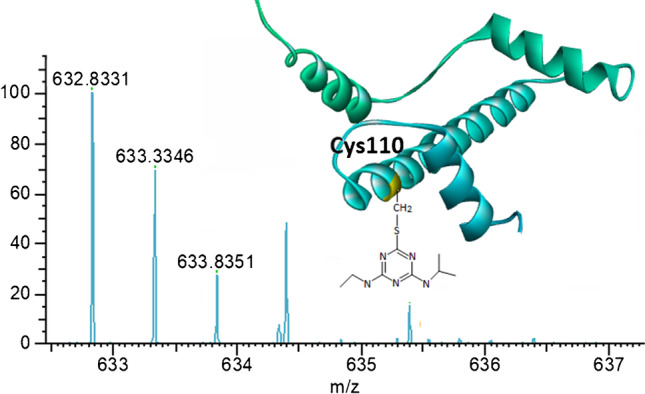

## Introduction

Proteins can covalently adduct with xenobiotic compounds from exposure to endogenous or exogenous chemicals, such as drugs, pesticides, or their metabolites, at active amino acid residues [1–8]. Research over the past half century has demonstrated that these protein adducts might lead to multiple health issues, including cancer and immune system effects [1, 5, 7, 9–13]. Therefore, identification of xenobiotics adducted to key proteins and identification of the sites of adduction within the protein are important to better understand the events underlying diseases and chemically induced adverse reactions. Generally, xenobiotic compounds can react with multiple proteins at multiple sites, and identification and characterization of adducted proteins are complicated and present significant analytical challenges [1, 14, 15].

Histones are small, basic, and highly conserved proteins. They are the major proteinaceous component of chromatin in eukaryotic cells and an important part of the epigenome. DNA is wrapped on the core of histones (formation of two H2A–H2B dimers and a H3–H4 tetramer), and the structure is stabilized by histone linker H1/H5 [16]. Histones affect most DNA-related events, including transcription, replication, and chromosome segregation, and play a key role in controlling DNA expression [14, 17–21]. Recent evidence has shown that alteration of histones by exogenous toxicants might conceivably disturb their normal function [14, 19, 22–25].

Atrazine (2-chloro-4-[ethylamino]-6-[isopropylamino]-1, 3, 5-triazine) is mainly used for control of broadleaf and grassy weeds, and it is one of the most commonly used herbicides in North America [26]. The primary health effects associated with atrazine exposure are reproductive and developmental abnormalities, while carcinogenesis data remain inconclusive [26]. The metabolic activation of atrazine is now relatively well understood, but the events that link exposure to atrazine to toxic sequelae remain ill-defined [27]. It was previously demonstrated that atrazine and its metabolites might form adducts on cysteine residues on hemoglobin and albumin in vitro or in vivo [28–31]. However, to our knowledge, there has been no research on the formation of atrazine adducts with histones.

As atrazine and its metabolites can form adducts with nucleophiles, such as free Cys, in hemoglobin and albumin proteins, we hypothesized that the nucleophile residues in calf thymus whole histones (mixture of histones) and human histone H3.3 are also targets of atrazine. The purposes of this research were to investigate possible non-enzymatic covalent adduction of histone with atrazine in vitro, and to characterize adducts formed and the specific amino acids modified.

## Materials and methods

### Chemicals and reagents

Calf thymus whole histones and human histone H3.3 (expressed in *E. coli*) were purchased from Millipore-Sigma (Oakville, ON, Canada). Sequencing-grade modified trypsin and Glu-C were purchased from Promega (Madison, WI, USA). Atrazine (analytical standard grade) was purchased from Sigma-Aldrich (Oakville, ON, Canada). A concentration of 0.5 M phosphate-buffered saline (PBS, pH = 7.4) was purchased from Fisher Sci. (Ottawa, ON, Canada). HPLC-grade acetonitrile (ACN), dimethyl sulfoxide (DMSO), ammonium bicarbonate (NH_4_HCO_3_), formic acid, and a BCA protein assay kit were from Millipore-Sigma. Water was purified on a Milli-Q system (Millipore, Billerica, MA, USA). Asn-Asn-Asn peptide (Asn_3_) was synthesized by Biomatik Corporation (Kitchener, ON, Canada).

Atrazine spike solution was prepared by dissolving an amount of atrazine in DMSO and stored at 4 °C until further use. Calf thymus whole-histone solution was prepared freshly on the day of study by dissolving an amount of histone in 50 mM PBS (pH = 7.4).

### Sample preparation and pre-treatment

For calf thymus histone (mixture of histones) adduction assay, 1 µL of 10 mg/mL atrazine DMSO solution was spiked into 500 µL of 1 mg/mL histone PBS solution (50 mM, pH = 7.4) and then the mixtures were incubated at 37 °C for 24 h under gentle shaking in a water bath. After incubation, 100 µL of the solution was taken and 400 µL ACN (−20 °C) was added to the sample, and vortexed well to quench the reaction. Then the sample was kept in a freezer (−20 °C) for half an hour. After that, the sample was centrifuged at 16,000 g for 10 min at 4 °C to precipitate the histone. The supernatant fraction was discarded, and the residue histone was washed three times with 200 µL of cold ACN (−20 °C) and subjected to centrifuge separation to remove unbound atrazine in the solution. The histone pellet was resuspended in 100 µL of 50 mM ammonium bicarbonate (NH_4_HCO_3_) buffer before enzymatic digestion.

For the human histone H3.3 adduction assay, since the commercial available H3.3 was supplied with buffer solution (1 mg/mL) and there were DTT and EDTA in the product, it was necessary to wash out DTT and EDTA before the adduction assay. A volume of 400 µL ACN (−20 °C) was added to 100 µL of 1 mg/mL human histone H3.3 solution and vortexed well; then, the sample was kept in a freezer (−20 °C) for half an hour. After that, the sample was centrifuged at 16,000 g for 10 min at 4 °C to precipitate histone. The supernatant was discarded, and the residue was washed with 200 µL of cold ACN (−20 °C) once. A volume of 100 µL of 20 µg/mL atrazine PBS solution (50 mM, pH = 7.4) was added to the residue. After that, the adduction assay was the same as for the calf thymus histone adduction assay previously described.

For the time-dependent adduct formation study, a set of calf thymus histone solutions was prepared as described above (500 µL of 1 mg/mL histone in PBS buffer), and spiked with the same amount of atrazine (1 µL of 10 mg/mL atrazine DMSO solution) and incubated at 37 °C under gentle shaking. At defined time points (0, 4, 8, 16, and 24 h), 100 µL of sample was taken and treated as described above.

For the concentration-dependent adduct formation study, a set of calf thymus histone solutions was prepared as described above (1 mg/mL histone in PBS buffer). Different concentrations of atrazine DMSO solutions (0, 2.5, 5, 7.5, and 10 mg/mL) were prepared, and 1 µL of the solution was spiked into 500 µL of 1 mg/mL calf thymus histone PBS solution (50 mM, pH = 7.4). The samples were incubated at 37 °C for 24 h under gentle shaking in a water bath. After that, 100 µL sample was taken and treated as described above. Histone incubated, without atrazine, under the same conditions (24 h at 37 °C) was used as the control. All the incubations were carried out in triplicates.

### Enzymatic cleavage

The histone adduction assay samples were digested by spiking 20 µL of 0.1 µg/µL trypsin (in 50 mM NH_4_HCO_3_) and 20 µL of 0.1 µg/µL Glu-C (in 50 mM NH_4_HCO_3_) into the sample and incubated at 37 °C overnight (16 h). The digestions were quenched by the addition of 10 µL of 1% formic acid. After centrifugation (5 min at 10,000 g), the supernatant was transferred to an LC vial for further analysis.

### Liquid chromatography–quadrupole Exactive-Orbitrap mass spectrometry analysis

Following the digestion of samples, the peptides present were analyzed using a Vanquish ultra-high-performance liquid chromatography interfaced with a quadrupole Exactive-Orbitrap mass spectrometer (UHPLC-Q-Exactive-Orbitrap-MS; Thermo Fisher Scientific, Mississauga, ON, Canada). Chromatographic separation was performed on a Kinetex XB-C18 column (100 × 2.1 mm, 1.7 µm particle size, Phenomenex Co., CA, USA). The mobile phase consisted of water (A) and ACN (B) both containing 0.1% formic acid. A volume of 100 µL of 10 mg/L Asn_3_ solution was spiked into each 1 L of mobile phase to create internal lock mass [32]. The elution conditions were as follows: 0–1 min, 5% B; 1–50 min, 5–40% B; 50–51 min, 40–95% B; 51–65 min, 95% B; 65–66 min, 95–5% B; and 66–76 min, 5% B (column re-equilibration). The injection volume was 5 μL, the flow rate was 300 µL/min, and the column was maintained at 40 °C.

The UHPLC-Q-Exactive-Orbitrap-MS was operated in full-scan mode and data-dependent acquisition fragment analysis (dd-MS2/dd-SIM, DDA) mode for identification. For the time-dependent adduct formation study and the concentration-dependent adduct formation study, the samples were analyzed in full-scan mode.

The UHPLC-Q-Exactive-Orbitrap-MS was performed in positive polarity (ESI ( +)) with a scanning range of 150 to 2000 m*/z*. Ion source parameters consisted of 3 K spray voltage, S-lens RF level of 60, 50 sheath gas flow rate, 20 auxiliary gas flow rate and zero sweep gas flow rate, 350 °C capillary temperature, and 500 °C for the auxiliary gas heater temperature.

In full-scan mode, the MS resolution was 70,000, AGC target was 3 × 10^6^, and maximum IT was 200 ms. In data-dependent mode (DDA), automatically switching between MS and MS/MS acquisition for the top 5 most abundant ions in each MS scan was selected for fragmentation in the HCD cell. A value of 28% normalized collision energy (NCE) was used to generate MS/MS spectra. MS/MS scans were performed with 17,500 resolving power, and with an AGC target value of 1 × 10^5^ and a maximum IT value of 50 ms. The isolation window and the dynamic exclusion value were 1.5 m/z and 4 s, respectively. Lock mass was 361.14662 m*/z* (Asn-Asn-Asn).

### UHPLC–Q-Exactive-Orbitrap-MS data preprocessing

For identification of histone adducts with atrazine, the acquired UHPLC–Q-Exactive-Orbitrap-MS raw data files were converted to MGF files using Raw Converter software. LC–MS data was then preprocessed with the open-source software ProteinProspector to search for histone adducts with atrazine. Taking into consideration that under ESI ionization multicharged ions were obtained for peptides, only ions with charges of + 2 and + 3 were selected. The mass tolerance was set to 5 ppm for precursor and 10 ppm for fragment ions. Trypsin/Glu-C was specified as the cleavage enzyme and maximum missing cleavage was set at 1. Methionine oxidation was specified as variable modifications. In the “User Defined Variable Modifications” parameter, the elemental composition of C_8_H_13_N_5_ (179.1171 m*/z*) from atrazine was selected for potential adduct to amino acid residue of Cys.

For the time-dependent adduct formation study and the concentration-dependent adduct formation study, the acquired UHPLC–Q-Exactive-Orbitrap-MS raw data (in full-scan mode) was analyzed by TraceFinder 5.0 (Thermo Fisher Scientific, Mississauga, ON, Canada). The potential atrazine-modified peptide and non-modified peptide ions were selected as target ions, and the peak areas in extracted chromatograms were compared.

## Results and discussion

### Sample preparation and pre-treatment

Protein purification ahead of digestion and analysis by UHPLC–Q-Exactive-Orbitrap-MS are front-end preparation strategies for proteome analysis. There are a variety of methods available to isolate proteins in proteomic and metabolomic workflows, which includes ultrafiltration, precipitation, and chromatographic methods. In our previous project, ultrafiltration was successfully used in the separation and concentration of albumin for adduction assays [30]. However, as the molecular weight of histones is much smaller than that of albumin, a low-molecule cut ultrafiltration kit had to be used (Vivaspin™ 500 centrifugal concentrators VS0112 (cut-off 5 kDa), from Sartorius Stedim North America, Inc., New York, NY, USA). This resulted in time-consuming and variable recoveries.

Precipitation is a classic approach that can be used to fractionate, concentrate, or purify low-molecular-weight proteins like histones from complex biological systems ahead of mass spectrometry analysis [33–36]. It was demonstrated that by combining the use of salt and acetone exceptional recoveries could be achieved for solvent-based protein precipitation. Several comparative studies examining the efficiency of protein precipitation have concluded that acetone precipitation provides the highest and most consistent recovery over alternative organic solvents [35]. However, acetone may modify protein or peptide structures and it was reported that a trace amount of residual acetone in the precipitated protein could lead to a significant number of peptides being modified following mass spectrometric analysis [36]. Therefore, in our project, ACN was used as organic solvent for precipitation of histone [34]. The recovery of calf thymus histone (mixture of histones) in our assay was 61 ± 3%, which was determined through the BCA assay (as total amount of protein), after precipitation and three wash cycles.

### Enzymatic cleavage

The vast majority of proteomics experiments to date rely on a bottom–up workflow approach where proteins are digested into peptides that can be efficiently analyzed using a wide range of LC–MS or MALDI-TOF–MS instruments. In bottom–up proteomic strategies, protein digestion is the most crucial step and has a large influence on the quality of protein identification [37]. Many proteases are available for this purpose, each having their own characteristics. Although trypsin is widely applied in bottom–up proteomics, our preliminary studies showed that the use of trypsin was not suitable for quantification of histone adduction in this study. For Lys- and Arg-rich domains in histone, tryptic peptides were too short or hydrophilic to be separated by UHPLC and efficiently detected by Q-Exactive-Orbitrap-MS. For the potential adduction site of cysteine 110 thiol groups of histone H3, the tryptic peptide with a length more than 30 amino acids (from in silico digestion) was too large to be detected. Multiple-enzyme digestions, which include combined, parallel, or successive use of multiple enzymes, are a strategy to increase protein and proteome coverage. In our study, to identify histones in the commercial calf thymus histone mixture, parallel multiple-enzyme digestion was used. The histones underwent chemical propionylation and were then tryptically digested, followed by analysis by UHPLC–Q-Exactive-Orbitrap-MS, to improve sequence coverage [8, 38]. The histone was also digested by Glu-C in ammonium bicarbonate buffer and analyzed by UHPLC–Q-Exactive-Orbitrap-MS. Combining the UHPLC–Q-Exactive-Orbitrap-MS results from the two parallel multiple-enzyme digestions, five histones could be identified in the commercial calf thymus histone mixture via a protein sequence database search, which included H2B(P62808), H2A(P0C0S9), H3.1(P68432), H1.3(A7MAZ5), and H4(P62803). For identification of potential adducts of Cys110 in H3.1 (or human H3.3), the samples were digested by combined multiple-enzyme digestion of trypsin and Glu-C in ammonium bicarbonate buffer. In this way, the target peptide (DTNLCAIHAK) could be detected with high sensitivity by UHPLC–Q-Exactive-Orbitrap-MS and dissection of its adduction was possible. As H3.1 from calf thymus and H3.3 from humans have very similar sequence, the tryptic/Glu-C peptide of DTNLCAIHAK was their target peptide.

### Determination of histone–atrazine adducts using UHPLC–Q-Exactive-Orbitrap-MS

One of the major advances in proteomics technology over the past decade is the availability of highly sensitive and high-resolution mass spectrometry systems, which have provided powerful analytical tools for the complete structural characterization and quantification of drug–protein covalent adducts in complex biological systems [39]. In the present study, we used an internal lock mass approach with the peptide Asn_3_. This provided substantially greater accuracy in mass spectrometric analysis [32]. In our preliminary studies, we digested the proteins with trypsin directly [22] or after propionylation [8, 38]. There were no interesting adducts detectable in these assays. The potential adducted peptide of (R)FQSSAVMALQEACEAYLVGLFEDTNLCAIHAK(R) was a weak broad peak in the extracted ion mass chromatogram. As the thiol group of Cys is a powerful nucleophile, we hypothesized that Cys110 in H3 (both in H3.1 from calf thymus and H3.3 from human) are adduction targets of atrazine. After the samples were digested by proteases of trypsin and Glu-C in ammonium bicarbonate buffer, the target peptide (DTNLCAIHAK) and its atrazine adduct (DTNLC_(ATZ)_AIHAK) could be detected with high sensitivity by UHPLC–Q-Exactive-Orbitrap-MS.

**Fig. 1:**
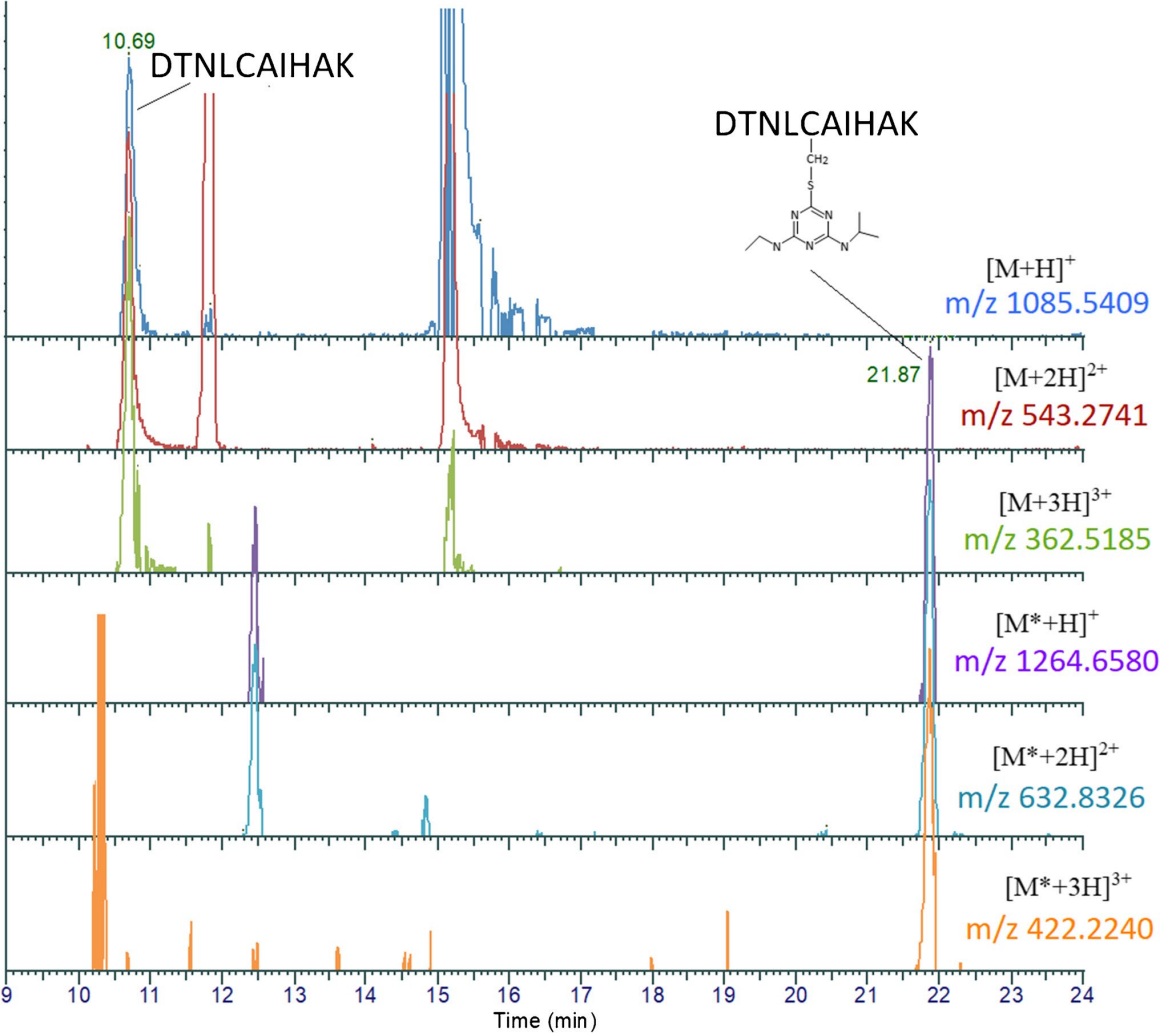
UHPLC-Q-Exactive-MS analysis and extracted ion chromatograms of the peptides DTNLCAIHAK [M] and DTNLC_(ATZ)_AIHAK [M*] from trypsin/Glu-C digested calf thymus histone, with adduct assay conditions of 20 µg/mL of atrazine and calf thymus histone solutions of 1 mg/mL histone in PBS buffer and an incubation time of 24 h

**Fig. 2:**
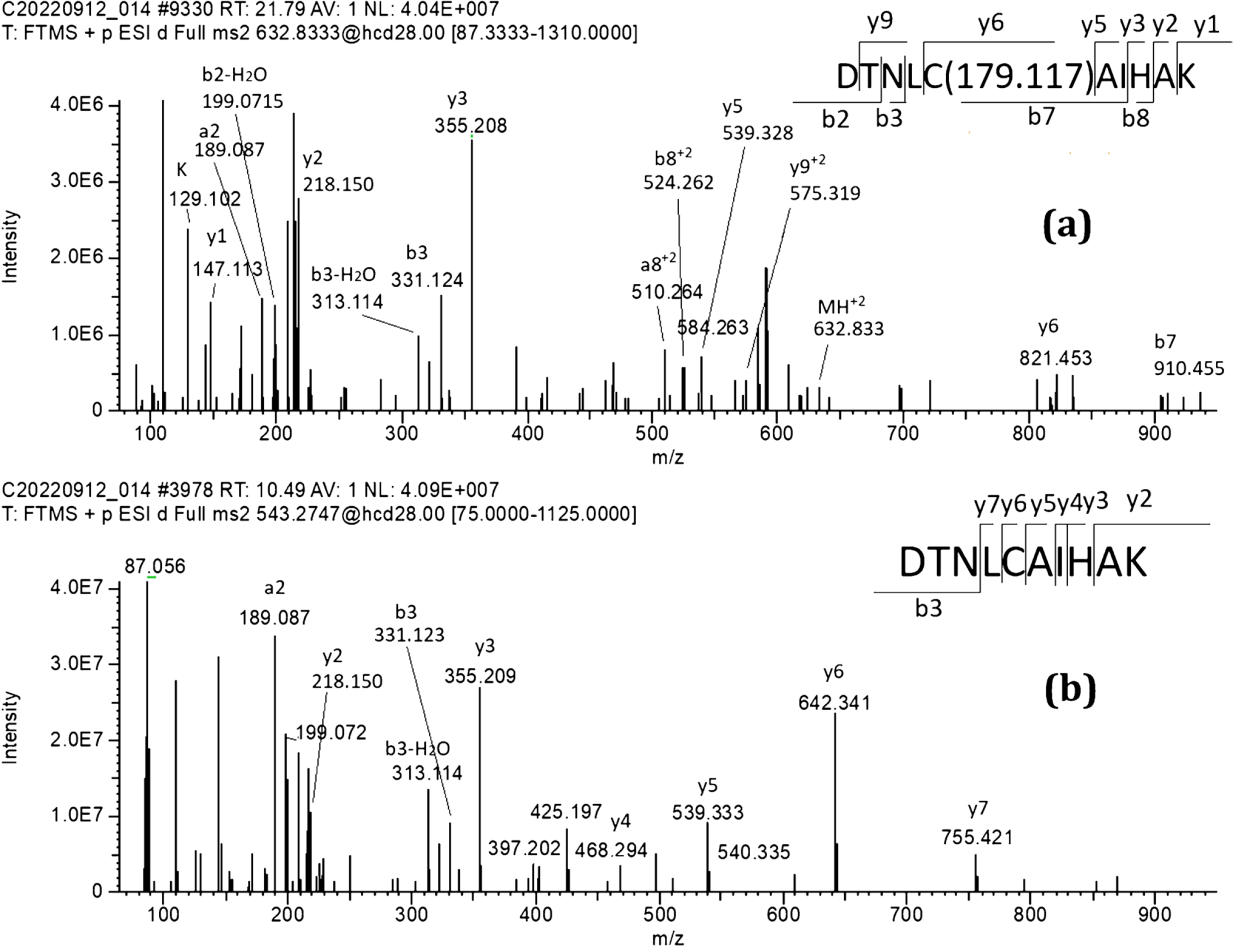
UHPLC-Q-Exactive-MS analysis and representative MS/MS spectrum of modified peptide of DTNLC_(ATZ)_AIHAK peptide (**a**) and unmodified DTNLCAIHAK peptide (**b**) from human histone H3.3. The MS/MS spectrums are that of the double-charged ion at *m/z* 632.833 and 543.275, corresponding to DTNLC_(ATZ)_AIHAK and DTNLCAIHAK peptide. This covalent modification was identified under adduction assay conditions of human histone H3.3 (1 mg/mL H3.3 in PBS buffer) incubated with 20 µg/mL of atrazine for 24 h and then digested by trypsin/Glu-C

**Fig. 3:**
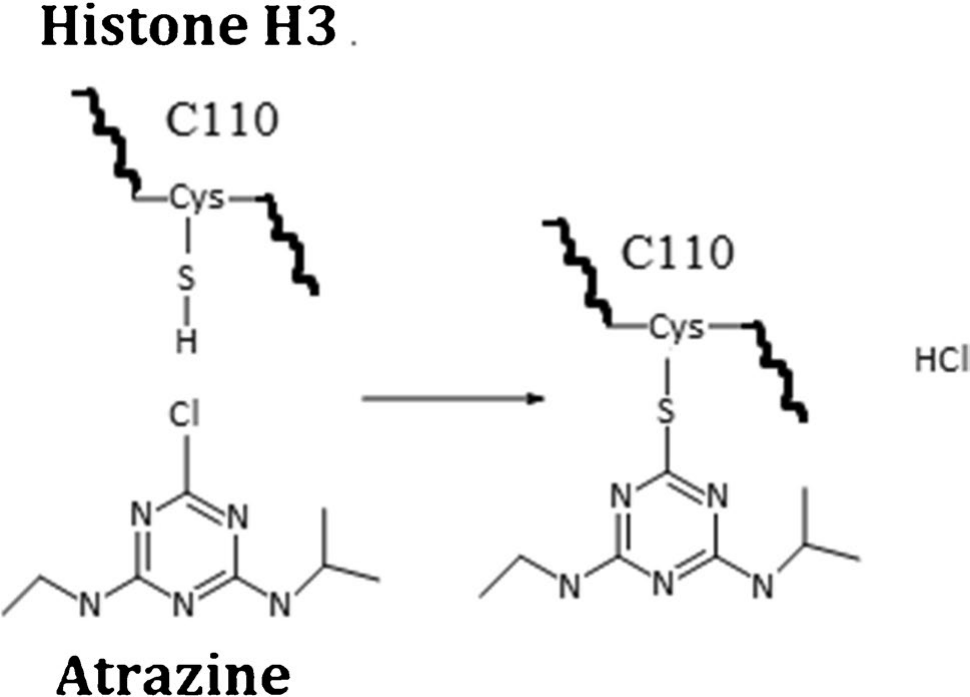
Adduct formation pathway of atrazine with histone H3

For the peptide DTNLCAIHAK, its [M + H]^+^, [M + 2H]^2+^, and [M + 3H]^3+^ (at *m/z* 1085.5409, 543.2741, and 362.5185, respectively) could be detected and separated from some interferences with the retention time of 10.7 min (Fig. 1). DTNLC_(ATZ)_AIHAK eluted at a retention time of 21.9 min. The ions of *m/z* 1264.6580, 632.8326, and 472.2240 corresponded to the single-, double-, and triple-charged peptide of DTNLC_(ATZ)_AIHAK, which showed an additional mass increment of 179.117 amu (C_8_H_13_N_5_) adduction (Fig. 1). The adduct was confirmed by the corresponding MS/MS spectrum, and the position of adduction at Cys110 was confirmed by the observation of the 179.1171 amu mass increment in the fragment ion y6 (*m/z* 821.4563) and subsequent ion of y9^2+^ (*m/z* 575.3192), while ions down to y5 (*m/z* 539.3301), y3 (*m/z* 355.2089), y2 (*m/z* 218.1500), and y1 (*m/z* 147.1129) did not display this increment (Fig. 2a, b). This confirmed that the atrazine covalent adduct was on Cys110 in the histone H3 sequence with the loss of HCl (Fig. 3).

**Fig. 4:**
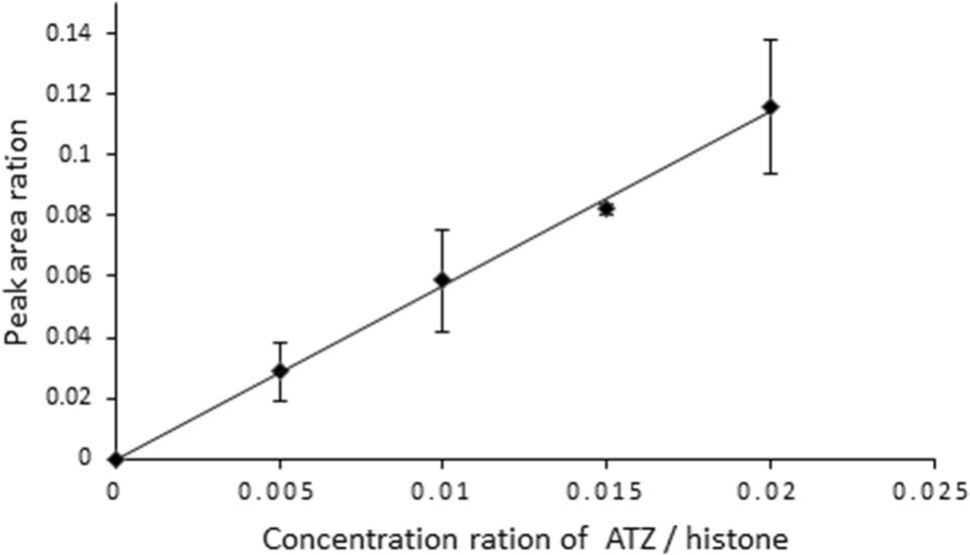
UHPLC-Q-Exactive-MS analysis and the profile of concentration-dependent adduct formation of calf thymus histone and atrazine. The peak area ratio is of the peak area in extracted ion chromatogram (EIC) of *m/z* 632.833 versus that of *m/z* 543.274. The incubation conditions were calf thymus histone solutions (1 mg/mL histone in PBS buffer) incubated with different atrazine concentrations (0 to 20 μg/mL), and at 37 °C for 24 h, and then digested by trypsin/Glu-C. All incubations were carried out in triplicate

**Fig. 5:**
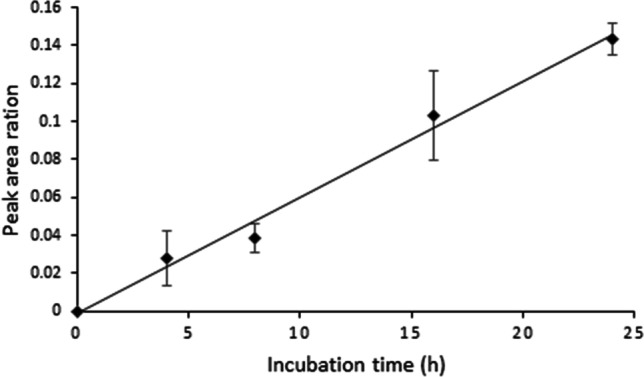
UHPLC-Q-Exactive-Orbitrap-MS analysis and the profile of incubation time-dependent adduct formation of calf thymus histone and atrazine. The peak area ratio is of the peak area in extracted ion chromatogram (EIC) of *m/z* 632.8326 versus that of *m/z* 543.2741. The incubation conditions were calf thymus histone solutions (1 mg/mL histone in PBS buffer) with 20 μg/mL atrazine concentration samples, and incubated at 37 °C for different times (0 to 24 h), and then digested by trypsin/Glu-C. All incubations were carried out in triplicate

The response intensity of the DTNLC_(ATZ)_AIHAK peptide showed a concentration-dependent increase in the histone atrazine adduction assay. The ratio of peak area in the extracted chromatogram of ions *m/z* 632.833 from DTNLC_(ATZ)_AIHAK versus that of *m/z* 543.274 from DTNLCAIHAK steadily increased from 0 to 0.14% with increasing concentration of atrazine from 0 to 20 µg/mL (histone concentration: 1 mg/mL and incubation time of 24 h, Fig. 4). Also observed was a time-dependent increase of the DTNLC_(ATZ)_AIHAK to DTNLCAIHAK peak area ratio in assay (Fig. 5). The peak area ratio in the extracted ion chromatogram of *m/z* 632.833 versus *m/z* 543.274 increased from 0 to 0.14% during incubation time from 0 to 24 h (incubation condition: 1 mg/mL histone and 20 µg/mL atrazine, Fig. 4).

## Conclusions

Histone H3 is the most extensively post-translationally modified among the five histones, and it is associated with gene activation [14]. The post-translational modification (PTM) offers a mechanism for regulating DNA transcription, replication, and repair [5]. The covalent modification of histones by chemical carcinogens or their metabolic electrophiles may provide relevant early compound-specific biomarkers of cancer. This is anticipated to be useful for accurate risk assessments to decrease the incidence of chemically induced cancers [19]. Using high-resolution UHPLC–Q-Exactive-Orbitrap-MS, the identified formation of histone H3 covalent adduct (H3.1 from calf thymus and H3.3 from human) with atrazine may provide a toxicologically relevant atrazine-specific biomarker of bioactivation. Since atrazine reacts with amine side chains present on the free Cys of histone, it can directly disrupt the electrostatic interaction with DNA or block canonical modifications on the same sites [19]. This approach method for identification of histone adduct can also open new avenues for the development of new compound-specific biomarkers of atrazine exposure.

## Data Availability

The mass spectrometry dataset analyzed for this study can be found in MassIVE with the dataset identifier MSV000090917.
